# Kinetics and stoichiometry of gallic acid and methyl gallate in scavenging DPPH radical as affected by the reaction solvent

**DOI:** 10.1038/s41598-022-12803-3

**Published:** 2022-05-24

**Authors:** Marzieh Sadat Shojaee, Marzieh Moeenfard, Reza Farhoosh

**Affiliations:** Department of Food Science and Technology, Ferdowsi University of Mashhad, Faculty of Agriculture, P.O. Box 91775-1163, Mashhad, Iran

**Keywords:** Biochemistry, Molecular biology, Plant sciences, Chemistry

## Abstract

The activity and capacity of gallic acid (GA) and methyl gallate (MG) in scavenging DPPH^·^ were determined in different solvents. Based on the bimolecular rate constants *k*_2_, both antioxidants showed highest activities in EtOH, followed by in MeOH, *t*-BuOH, MeCN, 2-PrOH, acetone, THF, ethyl acetate, and 1,4-dioxane. GA indicated better activities (*k*_2_ value, M^−1^ s^−1^) than MG in the alcoholic solvents (51–1939 vs. 25–1530) and in MeCN (203 vs. 187) whereas MG was of higher activities in the polar aprotic solvents (1.7–41 vs. 1.6–13). The highest stoichiometries for GA vs. MG were in 2-PrOH (6.67 vs. 5.37), followed by EtOH (5.84 vs. 4.57), MeOH (5.34 vs. 3.8) ~ acetone (5.02 vs. 4.44), MeCN (3.68 vs. 3.05) ~ *t*-BuOH (3.14 vs. 2.99), THF (2.34 vs. 2.2), ethyl acetate (1.2 vs. 0.93), and 1,4-dioxane (0.34 vs. 0.35).

## Introduction

According to a substantial body of evidence about the role of free radicals in fundamental cellular reaction, oxidative stress, and food products stability, a great deal of attention has been paid to the field of free radical chemistry in recent years. Nowadays, the increasing level of physical and mental stress, pollutions, and nutritional limitations have enhances risks of the generation of free radicals which cause chronic diseases such as Alzheimer's and diabetes, and carcinogenic diseases in biological systems. Also, the occurrence of free radicals in food systems is inevitable due to the biological nature of foods. Free radicals are mainly responsible for the initiation of the oxidation reaction in foods^1^.

Antioxidants play an essential role in both food systems and human body to reduce oxidative processes and the harmful effects of free radicals. In food systems, antioxidants retard lipid peroxidation and thereby help to protect the flavor, color, and texture of food products during storage. They can also protect human body by retarding the development of many chronic diseases and destructive reactions^2^. Potency of an antioxidant basically includes the two aspects activity and capacity, which are often used interchangeably^3^. Antioxidant activity deals with the kinetics of its inhibitory action, providing the reaction rate constant of an antioxidant with a specific oxidant like free radicals. Antioxidant capacity reflects stoichiometry, denoting the number of oxidant molecules effectively reduced by an antioxidant species^3,4^. DPPH (2,2-diphenyl-1-picrylhydrazyl) radical (DPPH^·^) assay, which is one of the popular methods to evaluate the kinetics and stoichiometry of antioxidative reactions, is commonly used due to its ease of use, speed and sensitivity^5^. The assay is based on the reduction of the purple chromogen DPPH^·^ by hydrogen atom or electron transfer from the scavenging molecule, i.e. antioxidant, which causes the formation of the pale yellow hydrazine (DPPH_2_)^6^.

Among natural constituents, phenolic compounds are known for their antioxidant potencies by donating hydrogen atoms or transferring electrons^7^. Gallic acid (3,4,5-trihydroxybenzoic acid, GA) has been suggested to possess high activities and capacities^8,9^. Several studies have reported greater activity of GA than ascorbic acid, Trolox, caffeic acid, sinapic acid, and vitamin E in scavenging DPPH^·^^10–13^. From the stoichiometric point of view, each molecule of GA has been found to reduce up to six radicals of DPPH^14^. Similarly, researchers have demonstrated that GA derivatives behave as highly potent DPPH^·^ scavengers with methyl gallate (methyl 3,4,5-trihydroxybenzoate, MG) of higher effectiveness than others^15,16^.

The kinetic analyses of the reduction of DPPH^·^ with many phenolic compounds in the two recent decades have suggested two different mechanisms for the reaction: (1) a direct hydrogen atom transfer (HAT) from phenol (ArOH) (reaction 1), and (2) an single electron transfer (SET) from the low concentration of preformed phenoxide anion (ArOˉ), present in equilibrium with ArOH, to DPPH^·^ (reaction 2)^17,18^. The two side reactions (3) and (4) of limited occurrence with the less reactive antioxidant radicals (ArO^·^) have been shown to be likely as well^19^.

1 $$ {\text{ArOH}} + {\text{DPPH}}^{\cdot} \to {\text{ArO}}^{\cdot} + {\text{DPPH}}_{{2}} $$

2 $$ {\text{ArO}}^{-} + {\text{DPPH}}^{\cdot} \to {\text{ArO}}^{\cdot} + {\text{DPPH}}^{-} \left( { + {\text{H}}^{ + } \to {\text{DPPH}}_{{2}} } \right) $$

3 $$ {\text{ArO}}^{\cdot} + {\text{DPPH}}^{\cdot} + \to {\text{ArO}} - {\text{DPPH}} $$

4 $$ {\text{ArO}}^{\cdot} + {\text{ArO}}^{\cdot} \to ({\text{ArO}})_{{2}} $$

Regardless of the innate potency of any antioxidants to scavenge DPPH^·^, the dominant reaction pathway and its rate has been shown to be remarkably affected by the nature of reaction solvent (e.g. permittivity, polarity, H-bond donating/accepting)^20,21^. Furthermore, it has been suggested that steric hindrance should be considered as a key point in DPPH^·^ assay because a molecular rotation is required for reactive groups in antioxidant molecules to orient towards the radical site in DPPH^·^^22^. Indeed, DPPH^·^ assay studies by taking simultaneously into account the two aspects solvent properties and steric accessibility will definitely give us better insight into the true potency of antioxidants.

Many researches are found in literature determining the capacity of antioxidants to scavenge DPPH^·^ with no addressing the kinetic studies in detail. Besides, the kinetic solvent effects as well as the steric accessibilities have not been considered in many of the kinetic studies during DPPH^·^ assay. Hence, the present study aimed to investigate the activity and capacity of GA and MG as efficient and common phenolic compounds in scavenging DPPH^·^ as affected by the reaction solvent and their steric accessibility to the radical site in DPPH^·^.

## Materials and methods

### Materials

GA, MG, and DPPH^·^ of analytical grade were purchased from Sigma-Aldrich (St. Louis, MO). All the solvents (Table 1) and other chemicals and reagents used in the study were of analytical grade and supplied by Merck (Darmstadt, Germany) and Sigma-Aldrich (St. Louis, MO).

**Table 1:**
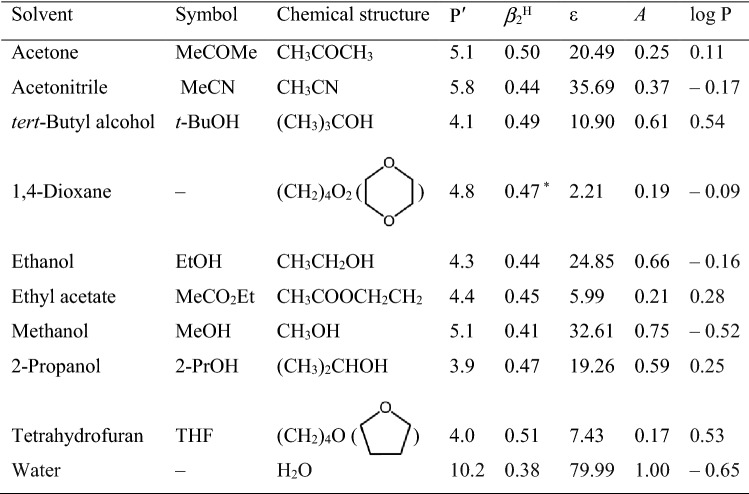
Selected reaction solvents in DPPH^·^ assay plus some of their physicochemical properties.

P′: Polarity index^23^; *β*_2_^H^: Relative hydrogen bond accepting (HBA) ability^24^; ε: Dielectric constant^25^; *A*: Anion solvating abilities of solvents^26^; log P: Octanol/water partition coefficient^27^; ^*^^28^.

### Preparation of DPPH– solutions

DPPH^·^ (0.0011 g) was dissolved in the reaction solvents for the preparation of stock solutions (60 µM). The solutions were prepared daily and used freshly. The exact initial DPPH^·^ concentration in the reaction medium was calculated spectrophotometrically (model 160A Shimadzu, Kyoto, Japan) from the calibration curves shown in Table 2.

**Table 2:** Calibration curve equations in triplicate as the absorbance (A) read at the wavelength of maximum absorption (λ_max_) versus the concentration (μM) of DPPH free radical in the different reaction solvents.

Solvent	Calibration curve equation	λ_max_ (nm)	R^2^
MeCOMe	A = 0.0107 [DPPH^·^] − 0.0030	519	1.000
MeCN	A = 0.0102 [DPPH^·^] − 0.0020	520	0.999
*t*-BuOH	A = 0.0105 [DPPH^·^] + 0.0006	518	0.999
1,4-Dioxane	A = 0.0100 [DPPH^·^] − 0.0078	519	0.999
EtOH	A = 0.0110 [DPPH^·^] − 0.0064	517	0.999
MeCOOEt	A = 0.0109 [DPPH^·^] − 0.0052	518	1.000
MeOH	A = 0.0108 [DPPH^·^] − 0.0021	517	0.998
2-PrOH	A = 0.0090 [DPPH^·^] + 0.0009	517	0.999
THF	A = 0.0095 [DPPH^·^] − 0.0073	517	0.999

### Preparation of antioxidant solutions

GA and MG were dissolved in the reaction solvents (Table 1) for the preparation of stock solutions (18–240 µM).

### Kinetic analysis

**Figure 1:**
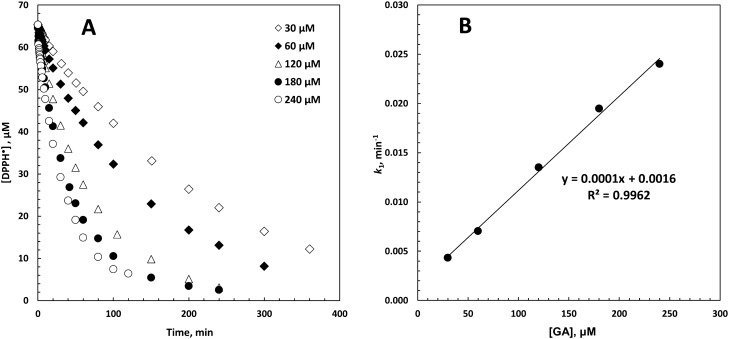
(**A**) The kinetic curve of [DPPH^·^] decay in the presence of gallic acid (GA) in 1,4-dioxane. (**B**) The pseudo-first-order rate constant *k*_1_ as a function of GA concentration in 1,4-dioxane.

In large excess concentrations of an antioxidant^29^, the decay of DPPH^·^ over time *t* is analyzed as a pseudo-first-order process according to Eq. (5):

5 $$ {\text{[DPPH}}^{ \bullet } {] } = {\text{ [DPPH]}}_{{0}} \, . \, e^{{ - k_{1} t}} $$

where [DPPH^·^]_0_ is the radical concentration at *t* = 0, and *k*_1_ is the pseudo-first-order rate constant (Fig. 1A). The slopes of the linear plots of *k*_1_ versus the concentration of the antioxidants provided the bimolecular or second-order rate constants *k*_2_ (Fig. 1B)^19^.

On the basis of the concentration and reactivity of an antioxidant, the kinetic curves of DPPH^·^ decay have revealed at least five different patterns of reactivity as shown in Table 3^22^. They are also differentiated from each other by the distinct ranges of the initial reaction rate R_i_ (nmol DPPH^·^ s^−1^) and the steric accessibility criterion (Table 3). The initial reaction rates R_i_ at the same concentration (60 μM) of DPPH^·^ and the antioxidants were calculated from the initial part of the exponential curves (Eq. 1), being determined by the first derivative (d[DPPH^·^]/d*t*) of Eq. (5) at *t* = 30 s:

6 $$ {\text{R}}_{{\text{i}}} = - {\text{[DPPH}}^{ \bullet } {]}_{{0}} \, {. }k_{1} . \, e^{{ - k_{1} t}} $$

**Table 3:** The different reactivity patterns of antioxidants in scavenging DPPH^·^ plus the corresponding ranges of their initial reaction rate and the steric accessibility criterion^22^.

Group	Initial [DPPH^·^] drop over time	R_i_ (nmol DPPH^·^/s)	ΔDPPH_f_/ΔDPPH_i_ ratio
Extremely fast	Immediate and the reaction completed in < 10 s	> 20.0	1
Fast	Rapid and the reaction continued more slowly	4.0–10.0	1.0–1.76
Medium	Slow and continuous	0.5–4.0	1.6–8.6
Slow	Very slow and nearly linear	0.0–0.5–	3.3–3.7–
Nonreactive	Low or no drop	–	–

The ratio between the final [DPPH^·^] drop after 60 min of the reaction (ΔDPPH_f_ = [DPPH^·^]_60_ − [DPPH^·^]_0_) and the initial [DPPH^·^] drop at *t* = 30 s (ΔDPPH_i_ = [DPPH^·^]_30_ − [DPPH^·^]_0_) was used to check the steric accessibility of the antioxidants.

### Stoichiometry of antioxidant reactions

After 60 min of DPPH^·^ decay (Table 1), the radical scavenging activity (RSA) was calculated as the percentage of DPPH^·^ bleaching using Eq. (7):

7 $$ {\text{RSA}}\;(\% ) = \left( {\frac{{A_{0} - A_{1} }}{{A_{0} }}} \right) \times 100 $$

where A_0_ and A_1_ correspond to the absorbances in the absence and presence of the antioxidants, respectively^30^. The concentration of the antioxidants required for scavenging 50% of the initial DPPH^·^ concentration (IC_50_) was calculated from the regression analysis of the response curve of RSA ($\mathrm{\%}$) as a function of the antioxidant concentration (μM). The stoichiometry value of the reaction (*n*), representing the number of the mole of DPPH^·^ reduced by one mole of antioxidant, was calculated by Eq. (8).

8 $$ n = \frac{{{\text{[DPPH}}^{ \cdot } {]}_{{0}} }}{{{2} \times {\text{IC}}_{{{50}}} }} $$

### Statistical analysis

All experiments and measurements were carried out in triplicate and data were subjected to analysis of variance (ANOVA). ANOVA and regression analyses were performed according to SPSS Statistics 22, SlideWrite version 7.0, and Excel 2013 software. Significant differences between means were determined by Duncan’s multiple range tests. P values less than 0.05 were considered statistically significant.

## Results and discussion

### Kinetic reaction patterns and initial reaction rates

**Figure 2:**
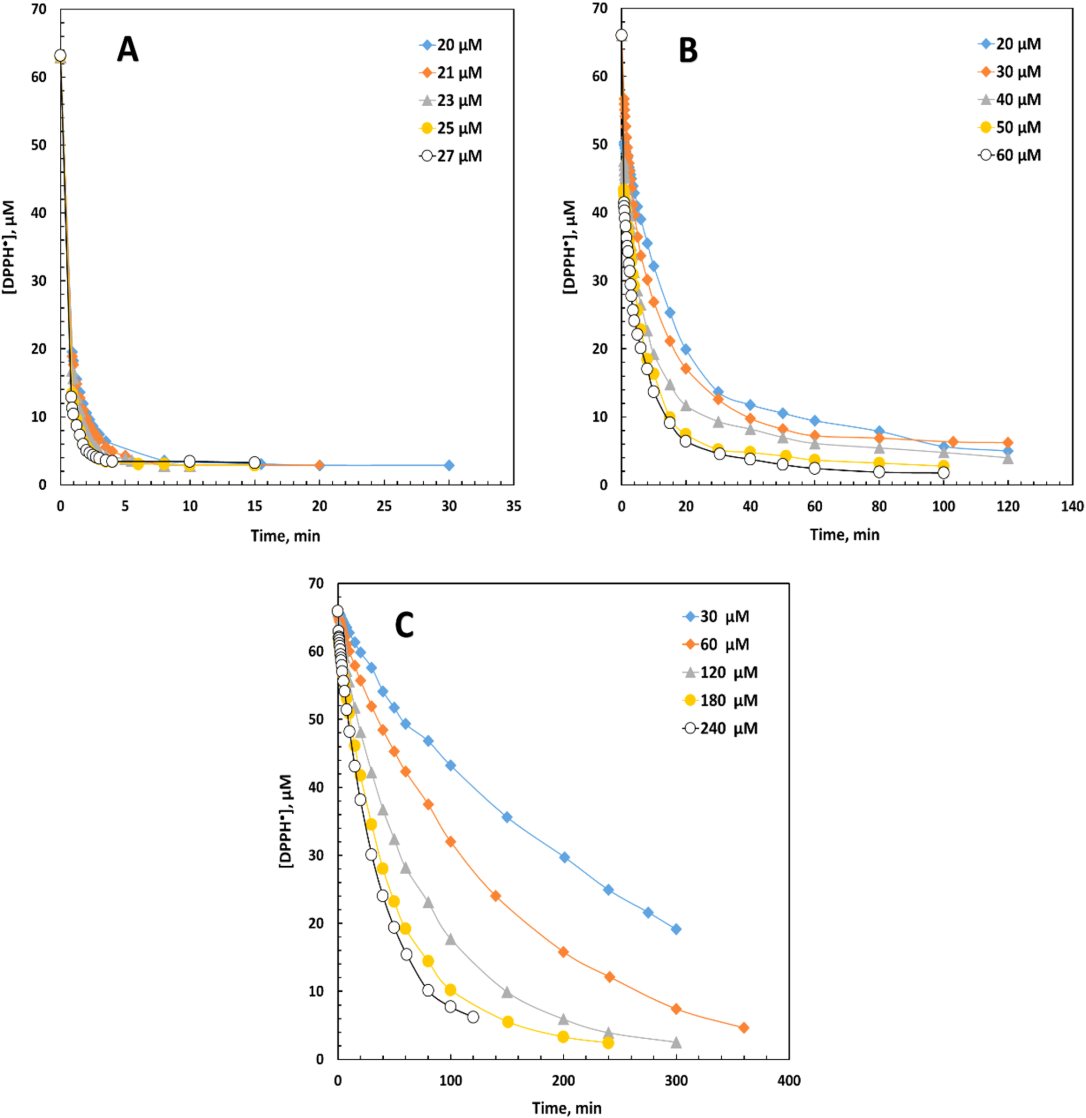
The kinetic curve of [DPPH^·^] decay in the presence of gallic acid (GA) in (**A**) methanol, (**B**) 2-propanol, and (**C**) 1,4-dioxane.

In the first step, the reaction kinetics of GA and MG were investigated separately in each solvent by monitoring DPPH^·^ decay under the pseudo-first-order conditions until a steady state was attained. Considerable variations in the shape of the reaction curves were observed in different reaction solvents (Fig. 2). According to Xie and Schaich^22^ (Table 3), the kinetic reaction patterns of different initial reaction rates (Table 4) were as the following groups:

- **Fast** GA and MG in MeOH (Fig. 2A), EtOH, and *t*-BuOH with rapid initial DPPH^·^ drops within a few minutes and then progressed more slowly in ~ 20–30 min.
- **Medium** GA and MG in 2-PrOH and acetone, respectively (Fig. 2B), and both antioxidants in MeCN. A continuous initial DPPH^·^ drop was observed until about one hour after the start of the reaction in 2-PrOH and acetone. The reaction in MeCN was faster, and the initial DPPH^·^ drop lasted for ~ 15 min.
- **Slow** GA and MG in ethyl acetate, 1,4-dioxane (Fig. 2C), and THF, GA in acetone, and MG in 2-PrOH. At low concentrations of the antioxidants in ethyl acetate and 1,4-dioxane, the initial [DPPH^·^] decreased for more than 4 h, and at higher concentrations, it continued for 2 h. The time was slightly shorter in THF, acetone, and 2-PrOH, and the initial DPPH^·^ drop was observed after ~ 1 h of the reaction time.

### Steric accessibility

The specific structure of DPPH^·^ and phenolic compounds may act as a barrier against each other and prevent reaching the phenolic OH groups to the radical site in DPPH^·^ due to steric hindrance, and then reduce the reactivity of antioxidants. In other words, steric accessibility to the radical site in DPPH^·^ plays a critical role in the radical scavenging capabilities of antioxidants. The ratio ΔDPPH_f_/ΔDPPH_i_ provided helpful information about steric accessibility to the radical site in DPPH^·^ (Table 4). Xie and Schaich^22^ reported that small monophenols with only hydroxyl ring adducts exhibit a ΔDPPH_f_/ΔDPPH_i_ ratio of ∼ 1, illustrating complete reaction within seconds (Table 3). Meanwhile, as the number and complexity of ring adducts increase, the reaction slows down since molecules must rotate to orient reactive groups towards the radical site in DPPH^·^. Accordingly, the ratio increases with the number and complexity of ring adducts. Nenadis and Tsimidou^30^ reported that large molecules, i.e. bulky ring adducts and/or multiple ring molecules, as well as the small molecules bearing one or two methoxy groups (e.g. ferulic acid) can be considered as “hindered phenols”. As shown in Table 4, GA and MG had the ΔDPPH_f_/ΔDPPH_i_ ratios higher than one, even in the solvents with the highest reaction rates (MeOH, EtOH, *t*-BuOH, and MeCN). Probably, steric factors may control the reaction of the antioxidants and interfere with phenol access to the radical site in DPPH^·^.

### Antioxidant activity

The results indicated that the polar protic/aprotic solvents of two alcoholic and non-alcoholic groups (Table 1) exerted significant impacts on the interaction between the antioxidants and DPPH^·^. According to the linear relationship between the pseudo-first-order rate constant *k*_1_ and the antioxidant concentrations, the second-order rate constants *k*_2_ were calculated from the slope of the plots (Fig. 1). The goodness of fit was excellent (R^2^ ~ 0.99) for all sets of the data, and the final results are given in Table 4.

### Alcoholic solvents

The highest values of *k*_2_ were found in EtOH and MeOH, respectively (Table 4). This was in accordance with the findings of Foti et al.^17^ who explained when the reaction is carried out in a hydrogen bond donating (HBD) solvent, the slow H-atom abstraction from antioxidant by DPPH^·^ (HAT mechanism, reaction 1) becomes a marginal reaction path and the reaction takes place through the fast SET mechanism (reaction 2). The significantly greater rate of the SET mechanism is essentially relevant to the partial ionization of phenols^30,31^. The extent of phenol ionization depends on the phenol acidity as well as the bulk and molecular properties of the reaction solvent, which are in turn related to the solvent permittivity and its ability to solvate and stabilize anions, respectively. The solvent permittivity is characterized by dielectric constant (ε), and the anion solvating ability of a solvent is quantified by Swain’s parameter (*A*) (Table 1). The SET mechanism will be the predominant pathway in solvents of high ε and *A* values such as EtOH, MeOH, or water, supporting the ionization of phenols to ArOˉ and rapid SET^20,30^. Furthermore, it has been postulated that the molecules of polar protic solvents are able to regenerate the catechol structure of phenols by a nucleophilic attack, leading to additional transfer of H-atoms to DPPH^·^^32^.

Surprisingly, the *k*_2_ values in EtOH were significantly greater than those in MeOH of higher P′, ε and *A* values (Table 4), being expected to better support the phenol ionization. Such a discrepancy can be due to the fact that analytical EtOHs usually contain higher contents of water (ε = 79.99 and *A* = 1.00) that more strongly supports the phenol ionization. Besides, analytical MeOHs have been shown to have higher amounts of acidic impurities^17^, which naturally suppress the ionization of phenolic OH groups.

**Table 4:** The initial reaction rates (R_i_) at an equal (60 μM) concentration of DPPH^·^ and the antioxidants, the ratio between the final [DPPH^·^] drop after 60 min of the reaction and the initial [DPPH^·^] drop at *t* = 30 s, the second-order rate constants (*k*_2_), the antioxidant concentration required for scavenging 50% of the initial [DPPH^·^] (IC_50_), and the number of the mole DPPH^·^ reduced by one mole of antioxidant (*n*) for the reaction between DPPH^·^ and the antioxidant gallic acid (GA) or methyl gallate (MG) in the different reaction solvents.

Solvent	R_i_ (nmol DPPH^·^ s^−1^)		ΔDPPH_f_/ΔDPPH_i_ ratio		*k*_2_ (M^−1^ s^−1^)		IC_50_ (μM)		*n*	
	GA	MG	GA	MG	GA	MG	GA	MG	GA	MG
MeCOMe	0.32 ± 0.01^Bd^	0.55 ± 0.02^Ac^	31.9 ± 0.5^Ad^	17.5 ± 0.5^Be^	12.8 ± 0.1^Bg^	40.6 ± 0.9^Ae^	6.55 ± 0.06^Bf^	7.67 ± 0.07^Af^	5.02 ± 0.08^Ac^	4.44 ± 0.08^Bb^
MeCN	3.64 ± 0.06^Bb^	3.73 ± 0.01^Bb^	2.24 ± 0.07^Af^	2.19 ± 0.02^Af^	203 ± 6^Ad^	187 ± 7^Bd^	9.33 ± 0.02^Be^	11.4 ± 0.1^Ad^	3.68 ± 0.02^Ad^	3.05 ± 0.00^Bd^
*t*-BuOH	4.20 ± 0.06^Aa^	3.97 ± 0.03^Ba^	1.55 ± 0.08^Ag^	1.64 ± 0.01^Ag^	609 ± 14^Ac^	265 ± 8^Bc^	10.4 ± 0.2^Bd^	11.3 ± 0.1^Ad^	3.14 ± 0.03^Ae^	2.99 ± 0.03^Bd^
1,4-Dioxane	0.04 ± 0.00^Af^	0.04 ± 0.00^Af^	97.2 ± 0.3^Ab^	97.7 ± 0.3^Ab^	1.60 ± 0.01^Bi^	1.70 ± 0.02^Ai^	96.5 ± 1.1^Aa^	95.9 ± 0.8^Aa^	0.34 ± 0.01^Ah^	0.35 ± 0.00^Ag^
EtOH	4.19 ± 0.08^Ag^	4.10 ± 0.02^Ag^	1.49 ± 0.05^Aa^	1.26 ± 0.10^Aa^	1939 ± 63^Aa^	1530 ± 25^Ba^	5.50 ± 0.06^Bg^	6.79 ± 0.09^Ag^	5.84 ± 0.07^Ab^	4.57 ± 0.09^Bb^
MeCOOEt	0.09 ± 0.01^Aa^	0.10 ± 0.01^Aa^	73.5 ± 4.3^Ag^	70.5 ± 1.5^Ag^	5.00 ± 0.00^Bh^	5.83 ± 0.05^Ah^	27.2 ± 0.4 ^Bb^	34.7 ± 0.4^Ab^	1.20 ± 0.01^Ag^	0.93 ± 0.01^Bf^
MeOH	4.51 ± 0.65^Aa^	3.92 ± 0.07^Aa^	1.29 ± 0.19^Ag^	1.51 ± 0.02^Ag^	1647 ± 21 ^Ab^	1003 ± 8^Bb^	5.93 ± 0.27 ^Bfg^	8.59 ± 0.46^Ae^	5.34 ± 0.23^Abc^	3.80 ± 0.19^Bc^
2-PrOH	0.64 ± 0.02^Ac^	0.37 ± 0.01^Bd^	12.6 ± 0.2^Be^	21.6 ± 0.7^Ad^	51.3 ± 0.9^Ae^	24.8 ± 1.1^Bf^	5.02 ± 0.06^Bh^	6.24 ± 0.04^Ag^	6.67 ± 0.06^Aa^	5.37 ± 0.05^Ba^
THF	0.20 ± 0.01^Ae^	0.22 ± 0.01^Ae^	47.9 ± 1.1^Ac^	43.6 ± 0.7^Bc^	14.7 ± 0.1^Bf^	20.7 ± 0.4^Ag^	14.6 ± 0.1^Bc^	15.4 ± 0.1^Ac^	2.34 ± 0.01^Af^	2.20 ± 0.04^Be^

Means ± SD (standard deviation) within a column with the same lowercase letters are not significantly different at P < 0.05. Means ± SD (standard deviation) within a row for each kinetic parameter with the same uppercase letters are not significantly different at P < 0.05.

Another discrepancy was the quite higher *k*_2_ values for GA than for MG in both the polar protic solvents. This was while the Hammett sigma constants (σ_p_), as measures of how strongly ring substituents at *meta* and *para* positions donate or withdraw electrons from reactive groups, have been reported to be 0.00 and 0.45 for the carboxylate anion COO^–^ (resulting in lower acidity for the phenolic OH group) and COOH/COOMe groups, respectively^33^. In their study on the SET reaction of some cinnamic acids and their methyl esters with DPPH^·^ in MeOH and EtOH, Foti et al.^17^ observed higher activities for the esters, interpreted as self-suppression of phenol ionization by the COO^–^/COOH group. The greater activity of GA, therefore, might have been arisen from its stronger solvent-based dynamism to interact with DPPH^·^. In other words, more polar solvents (P′ and log P values in Table 1) are expected to establish more dynamic reaction environments in which more polar antioxidants are of relatively higher solubility as well as of more frequent collisions with DPPH^·^^34^. On this basis, the lower molecular hydrophobicity of GA (log P = 0.31) than MG (log P = 0.77)^35^ essentially provides a more homogenous chemical environment of closer polarity for GA to collide more with the radical.

The other two polar protic solvents, *t*-BuOH and 2-PrOH, of lower polarities and ε and *A* values (Table 1) provided smaller bimolecular rate constants, respectively, as well as similar patterns of antioxidant activity. In fact, these solvents showed to be less supportive than EtOH and MeOH to ionize phenols and therefore to the rapid ET. Also, 2-PrOH caused more steric hindrance than *t*-BuOH in the accessibility to the radical site in DPPH^·^ (ΔDPPH_f_/ΔDPPH_i_ ratios in Table 4). Interestingly, the non-alcoholic solvent MeCN of relatively high polarity and dielectric constant (P′ = 5.8 and ε = 35.69, Table 1) provided partly high values of the second-order rate constant. MeCN has been shown to support phenol ionization to a great extent^36^. In addition, the antioxidants in MeCN had the ΔDPPH_f_/ΔDPPH_i_ ratios very close to those in the alcoholic solvents MeOH and EtOH (Table 4).

### Non-alcoholic solvents

The lowest values of *k*_2_ were found in 1,4-dioxane and ethyl acetate, respectively (Table 4), with very small quantities of the permittivity and Swain’s parameter (Table 1). The solvents with low ε and *A* values (e.g. alkanes, ε = 1.8, *A* = 0.00) have been shown to govern the dominance of the HAT mechanism^20,26,37^. Polar aprotic solvents are capable of accepting hydrogen bonds from phenols and then impede the H-atom transfer due to steric hindrance. Therefore, the HAT mechanism can only occur from the phenol fraction that is not H-bonded^17,30^. The significantly higher values of *k*_2_ in ethyl acetate than in 1,4-dioxanne indicate higher contributions of the antioxidant molecules reacting with the radical in the former. This can be confirmed by the lower Abraham et al.’s *β*_2_^H^ value^24^ (Table 1) for ethyl acetate (0.45 vs. 0.47), a measure of hydrogen bond accepting (HBA) ability of solvents on a relative scale from 0.00 to 1.00. Moreover, ethyl acetate with a higher dielectric constant (ε = 5.99 vs. 2.21) has been shown to be likely to partially support phenol ionization in some cases^28^. The significantly higher ΔDPPH_f_/ΔDPPH_i_ ratios for GA and MG in 1,4-dioxane (97.2 and 97.7, respectively) than in ethyl acetate (73.5 and 70.5, respectively) can also provide an additional explanation for the reduced reactivity of the antioxidants in 1,4-dioxane (Table 4).

By contrast with the relative activities of GA and MG in the alcoholic solvents, MG turned out to be of significantly faster H-atom transfer to DPPH^·^ in the polar aprotic solvents ethyl acetate and 1,4-dioxane (Table 4). This is apparently inconsistent with the σ_p_ values of COO^–^/COOH (0.00/0.45) and COOMe (0.45) groups^34^, so that the proton dissociation of COOH group generates COO^–^ group of higher electron donating effect, leading to a lower value of the phenolic O–H bond dissociation enthalpies (BDE) in GA^38^. More powerful antioxidants have lower O–H BDE, facilitating the direct H-atom transfer to a radical^39^. However, the O–H BDE values of GA and MG have been calculated to be 91.98 and 91.70 kcal mol^−1^ in gas phase, 91.68 and 91.67 kcal mol^−1^ in the nonpolar solvent benzene, and 91.06 and 90.42 kcal mol^−1^ in the polar aprotic solvent acetone, respectively^40^. The decreased O–H BDEs from the values in gas phase to those in acetone imply the solvating effect of acetone through its intermolecular H-bonding with the phenolic OH groups, which is greater on MG (91.70–90.42 = 1.28 kcal mol^−1^) than on GA (91.98–91.06 = 0.92 kcal mol^−1^). This means that MG donates H-atom more easily than GA in the polar aprotic solvents. The same relative activity pattern can also be observed for MG versus GA in acetone (40.6 vs. 12.8 M^−1^ s^−1^) and THF (20.7 vs. 14.7 M^−1^ s^−1^) of lower ΔDPPH_f_/ΔDPPH_i_ ratios (17.5 vs. 31.9 in acetone, 43.6 vs. 47.9 in THF) (Table 4). The higher *k*_2_ values of course arise from their supporting phenol ionization to more extent^36^.

### Antioxidant capacity

In addition to the rate at which an antioxidant reacts with DPPH^·^, the stoichiometry of the reaction is of crucial importance to generally evaluate radical scavenging potencies. Provided adequate time to scavenging, the maximum number of the moles of DPPH^·^ reduced by one mole of an antioxidant depends essentially on the fraction of the antioxidant molecules being able to react with the radical. This fraction is undoubtedly affected by the physicochemical properties of the reaction solvents, which may significantly affect the extent of phenol ionization^17^, steric accessibilities^22^, and regeneration of the phenolic structure leading to additional H-atom transfers^33^. That is, the reaction solvent may remarkably change innate potency of an antioxidant to scavenge radicals on a molar scale.

The capacity of GA and MG to reduce DPPH^·^ in terms of the IC_50_ or *n* values is shown in Table 4. The highest capacities of the antioxidants, on the whole, were obtained in 2-PrOH, followed by EtOH, MeOH ~ acetone, MeCN ~ *t*-BuOH, THF, ethyl acetate, and 1,4-dioxane. Such an order demonstrates well the undeniably greater contribution of the protic than aprotic solvents of higher polarity and permittivity, of lower interference in the steric accessibilities, and of more capability to regenerate phenols (especially in 2-PrOH with *n* ~ 6.7 for GA capable of scavenging ≤ 6 DPPH^·^ according to the reactions 1–4) to the higher stoichiometries.

As can be seen in Table 4, GA was of significantly higher capacity than MG in reducing DPPH^·^, which was in agreement with other research findings^16,41^. This, similarly, might have been governed by potentially the higher extent of phenol ionizations/regenerations and/or lower steric accessibilities for GA. Significantly the same antioxidant capacities in the polar aprotic 1,4-dioxane can be ascribed to the suppression of phenols ionizations/regenerations as well as to their statistically similar ΔDPPH_f_/ΔDPPH_i_ ratios in the solvent.

## Conclusions

Antioxidative evaluations require simultaneous studying the activity and capacity, standing for the kinetics and stoichiometry, respectively, of an antioxidant in scavenging radicals. Theoretical evaluations in gas phase of typically no intermolecular relationships may provide some valuable information on the innate potency, encompassing the activity and capacity, of individual antioxidant molecules. On this basis, gallic acid and methyl gallate are considered to be of roughly the same antioxidant potencies. However, the antioxidants were clearly shown to have dramatically different comparative potencies as a function of the type and strength of their molecular interactions with the polar protic/aprotic solvents. For gallic acid and methyl gallate, the solvents studied caused a wide diversity in the phenol ionizations, steric accessibilities towards the radical site in DPPH^·^, dynamism to interact with DPPH^·^, and regeneration of the phenolic OH groups. Hence, regardless of the type of radical present in an oxidizing environment and many other complexities, the extrapolation of the antioxidant potencies arising from DPPH^·^ assays to those in a system of interest (e.g. lipids, emulsions, and biological media) at first step requires as high physicochemical similarities as possible in their reaction environments.

## Data Availability

All the necessary data generated and/or analysed during the current study are included in this published article and its additional information, if needed, are available from the corresponding author on reasonable request.
